# A Cross-Sectional Study To Assess Knowledge of Monkeypox Among Medical Students and Graduates in India

**DOI:** 10.7759/cureus.49744

**Published:** 2023-11-30

**Authors:** Ria Singh Rawat, Dinesh Ramasetty, Rajesh Yadavalli, Meghana Kakarla, Shourya Meyur, Nilashis Dutta, Shreya Deoghare

**Affiliations:** 1 Department of Internal Medicine, RajaRajeswari Medical College and Hospital, Bangalore, IND; 2 Department of Internal Medicine, Dr. Pinnamaneni Siddhartha Institute of Medical Sciences and Research Foundation, Vijayawada, IND; 3 Department of Internal Medicine, Rajiv Gandhi Institute of Medical Sciences, Adilabad, IND; 4 Department of Internal Medicine, Kempegowda Institute of Medical Sciences, Bengaluru, IND; 5 Medicine, AMA School of Medicine, Makati, PHL; 6 Internal Medicine, Sambhunath Pandit Hospital, Kolkata, IND; 7 Department of General Medicine, North Bengal Medical College and Hospital, Siliguri, IND; 8 Department of Dermatology, Dr. DY Patil Medical College and Hospital, Pune, IND

**Keywords:** online resources, twitter, instagram, medical students, monkeypox

## Abstract

Background and aim

Monkeypox outbreaks in several nations have brought focus to the emerging virus. The clinical presentation of monkeypox is less severe than smallpox but is fairly comparable, presenting with fever, headache, lymphadenopathy, back discomfort, myalgia, and skin rashes. The misinformation spread on social platforms had a major impact on the effectiveness of outbreak control measures. The clinical management of breakouts depends on the adequate knowledge of the healthcare personnel about the disease. This study aims to assess the knowledge of medical students and graduates regarding monkeypox in India.

Methods

A cross-sectional survey was conducted by circulating a predesigned questionnaire with 20 questions to collect information regarding the demographic characteristics of the study participants, source of knowledge about monkeypox disease, self-perceived knowledge, and number of correctly answered questions regarding the disease. The responses obtained from the questionnaire and teleconsultation were recorded and analyzed.

Results

Out of 404 medical students and graduates who participated in the study, the mean age of participants was 23.17±2.14 years, and only 156 students learned about monkeypox in medical colleges. Only 72 (17.82%) participants reported that their medical college or hospital organized an educational class to teach about monkey pox disease. A majority of respondents (n=350, 86.63%) received their knowledge from online sources, which were the first most used source, followed by Instagram.

Conclusions

The knowledge assessment about monkeypox revealed that the average correct responses of participants who perceived to be knowledgeable were significantly lower than those who perceived to have good knowledge.

## Introduction

A double-stranded DNA virus known as monkeypox is a member of the orthopoxviral genus and family [1]. This virus is considered a zoonotic pathogen because it affects a wide variety of mammalian species. In Central Africa, human infections are the most common [2]. After the eradication of smallpox, it has emerged as the primary orthopoxvirus that affects humans.

This virus was first described in humans in 1970 in the Democratic Republic of Congo [3]. Contact with wildlife reservoirs was the source of the sporadic illness, which has been widely recorded in Africa. Secondary dissemination has been very limited, making human-to-human transmission ineffective. The World Health Organization (WHO) declared monkeypox an "evolving risk of moderate global concern" on June 23, 2022, after more than 3000 infections with the monkeypox virus were found in more than 50 different nations since early May 2022. In July 2022, the WHO declared the monkeypox outbreak, which was spreading throughout the globe, as a "public health emergency of international concern" [4].

The monkeypox virus can spread through large respiratory droplets, intimate contact with skin lesions, direct contact, and potentially contaminated fomites. Additionally, vertical transmission from mother to fetus was described, leading to fetal fatalities.According to the WHO, men account for 99% of cases, and at least 95% of these individuals have had intercourse with other men [1,5].

Monkeypox has a milder clinical presentation than smallpox, but the infection's symptoms are often similar [5]. Fever, migraines, lymphadenopathy, back pain, myalgia, and skin rashes are among the symptoms. The rash typically appears on the extremities and starts as maculopapules, then progresses to vesicles, pustules, and ultimately crusts.

The aim of this study is to assess knowledge of monkeypox disease among medical students and graduates in India.

## Materials and methods

This is a cross-sectional type of observational study conducted over a period of seven days. Ethics Committee approval was obtained prior to starting the study by the Independent Ethics Committee with the protocol number ECG003/2022. 

A questionnaire (see appendix) was designed and made available online through the link generated using Google Forms (Google, Inc., Mountain View, US). The questionnaire contained information regarding the demographic characteristics of study participants, source of knowledge about monkeypox disease, self-perceived knowledge, and the number of correctly answered questions regarding monkeypox.

The voluntarily participating medical students who are pursuing their Bachelor of Medicine, Bachelor of Surgery (MBBS) degree and graduates who have earned their MBBS degree and are employed by a hospital or other medical institution in India were included in the study. People who refused to take part in the study were excluded from the study.

The responses obtained from the questionnaire and teleconsultation were recorded in Google Sheets (Google, Inc., Mountain View, US) and transferred to Microsoft Excel (Microsoft® Corp., Redmond, US). Statistical analysis was performed using R Studio Version 4.3.2. (R Foundation for Statistical Computing, Austria).

## Results

A total of 404 medical students, interns, and graduates participated in the study. The demographic characteristics of participants are presented in Table 1. The mean age of participants was 23.17±2.41 years. There were 210 (51.98%) males and 194 (48.02%) females in the study. Out of the total, 174 (43.07%) medical students, 68 (16.83%) interns and 168 (40.10%) graduates participated in the study.

**Table 1:** Demographic characteristics of participants * Age is represented as mean and standard deviation

Variable	N (%)
Age*	23.17 ± 2.41 years
Educational level	
Medical students	174 (43.07%)
Interns	68 (16.83%)
Graduates	168 (40.10%)
Gender	
Males	210 (51.98%)
Females	194 (48.02%)

Out of 404 participants, 156 (38.61%) reported that they read about monkeypox disease from textbooks suggested by medical school. Only 72 (17.82%) participants reported that their medical college or hospital organized an educational class to teach about monkeypox disease. 

Table 2 shows the resources from which participants read about monkeypox disease: textbook 34 (8.42%), newspaper 20 (4.95%), and online resources 350 (86.63%). 

**Table 2:** Resources from which participants read about monkeypox disease

Resource	N (%)
Textbook	34 (8.42%)
Newspaper	20 (4.95%)
Online resources	350 (86.63%)
Instagram	104 (25.74%)
UpToDate	38 (9.41%)
Facebook	12 (2.97%)
Twitter	10 (2.48%)
Linkedin	8 (1.98%)
WHO website	138 (34.16%)
Other websites	178 (44.05%)

To assess self-perception about knowledge of monkeypox disease, the participants were asked to evaluate their level of knowledge on a scale from 1 to 10. Those who evaluated their level from one to five were considered to have poor self-perceived knowledge about the disease (group A). Those who evaluated their knowledge from six to ten were considered to have good self-perceived knowledge about the disease (group B). Two hundred and six (50.99%) participants felt that they had poor self-perceived knowledge. One hundred and ninety-eight (49.01%) participants had good self-perceived knowledge. Table 3 shows the self-perceived knowledge about monkeypox by participants on a scale from 1 to 10, with 1 being poor knowledge and 10 being excellent knowledge. Most participants scored their knowledge with 5, 6, and 7.

**Table 3:** Self-perception of participants about their knowledge about monkeypox disease

Group	Score (1-10)	N (%)
Group A: poor self-perceived knowledge (n=206, 50.99%)	1	24 (5.94%)
	2	14 (3.47%)
	3	36 (8.91%)
	4	48 (11.88%)
	5	84 (20.79%)
Group B: good self-perceived knowledge (n=198, 49.01%)	6	78 (19.31%)
	7	72 (17.82%)
	8	34 (8.42%)
	9	8 (1.98%)
	10	6 (1.49%)

A set of 20 questions about monkeypox disease were asked. Table 4 shows the number of participants who answered correctly. 

**Table 4:** List of questions used to assess knowledge of monkeypox disease among participants and number of participants who correctly answered the questions

Question	Correct responses (N, %)
1. The first human case of monkeypox was discovered in 1970.	338 (83.66%)
2. The recent outbreak of monkeypox cases was seen in May 2022.	360 (89.11%)
3. In the recent outbreak, the initial cluster of cases was reported in the United States.	212 (52.48%)
4. Inoculation of the virus to skin and mucosal surfaces occurs by direct contact	324 (80.20%)
5. Monkeypox virus replicates at the site of inoculation	274 (67.82%)
6. The incubation period lasts from four to five days.	284 (70.30%)
7. During the incubation period, the monkeypox virus is contagious and highly infectious	312 (77.23%)
8. Manifestations of the monkeypox virus can be seen in the prodromal stage	268 (66.34%)
9. Monkeypox is a sexually transmitted infection	202 (50.00%)
10. The majority of reported monkeypox cases have a travel-related link to an endemic country	346 (85.64%)
11. Most monkeypox cases have been among people who identify as men who have sex with men	238 (58.91%)
12. Monkeypox patients also have a sore throat along with a fever	342 (84.65%)
13. Monkeypox patients present with skin lesions	390 (96.53%)
14. The vesicles are incredibly painful	276 (68.32%)
15. Many monkeypox patients present with genital pain, rectal pain	246 (60.89%)
16. Treatment of monkeypox is mostly symptomatic	352 (87.13%)
17. Two antiviral drugs that may be used for monkeypox infections are tecovirimat and brincidofovir	326 (80.69%)
18. Post-exposure vaccination can also be given to patients who have had close contact with an individual infected with the monkeypox virus	318 (78.71%)
19. Smallpox vaccination provides cross-protection against monkeypox	264 (65.35%)
20. Vaccination is given within four days of exposure to prevent disease or up to 14 days after exposure to reduce the severity of the disease	312 (77.23%)

The average test scores among correct answers were then compared in groups A and B (see Table 5). Participants in group A with poor self-perceived knowledge about monkeypox disease had an average test score (12.68±2.33) lower compared to participants in group B with good self-perceived knowledge (13.52 ± 2.34). This difference was tested by a two-sample t-test, and it was statistically significant (p<0.05).

**Table 5:** Comparison of average test scores of the two groups A and B

Self-perceived knowledge about monkeypox	Average test score	SD	P-value
Group A: poor self-perceived knowledge	12.68	2.33	0.0004
Group B: good self-perceived knowledge	13.52	2.34	

## Discussion

The ongoing monkeypox outbreaks in several nations have brought the emerging virus into focus. Clinical management of monkeypox outbreaks necessitates the close coordination of knowledgeable medical professionals. For all facets of disease treatment, a comprehensive knowledge of the illness is necessary. According to a study by Sallam et al., the majority of the participants entrusted information about the viruses from healthcare professionals and scientific experts [6]. Several studies emphasize the pivotal role of precise and timely information supplied by scientific experts, medical journals, and physicians in various aspects, such as identifying cases, administering vaccinations, and positively contributing to top-tier medical responses during infectious disease outbreaks [7,8]. Therefore, the purpose of the study was to examine the knowledge of the monkeypox virus among medical students, interns, and graduates from various medical colleges in India.

A total of 404 medical students, interns, and graduates participated in the study. Out of the total, 174 (43.07%) medical students, 68 (16.83%) interns and 168 (40.10%) graduates participated in the study. This was similar to a study that was conducted by Harapan et al. to evaluate the expertise of general practitioners in Indonesia, where a total of 432 general practitioners were included [3]. The mean age of our participants was 23.17±2.41 years, whereas in Harappan et al. study, 293 (67.8%) were 30 and below years old, and 139 (32.2) were above 30 years old. Both studies had a relatively young group of doctors involved in the study. Our study contained 210 (51.98%) males and 194 (48.02%) females. This was in contrast to the study conducted by Harappan et al. in Indonesia, where 67.7 % of respondents were women [3]. Our study has a nearly equal representation of doctors from both sexes.

In our study, the resources from which the participants read about monkeypox disease were textbooks (8.42%), newspapers (4.95%), online resources (86.63%), namely online web (34.16%), Instagram (25.74%), UpToDate (9.41%), Facebook (2.97%), Twitter (2.48%), LinkedIn (1.98%) and other websites (23.27%). This was comparable to a study done on medical students' perception of monkeypox by Alshahrani et al. in Saudi Arabia, where the informational source on monkeypox for the participants was Twitter (62.1%), Snapchat (48.1%), television (30.8%), and WhatsApp groups (16.2%), friends and relatives (4.2%) and research articles (14.7%) [9]. Online resources played a major role in providing information about monkeypox to participants of both studies. However, misunderstandings in analyzing medical data acquired online might result in a lack of health knowledge and/or online literacy. A suitable education program and assessment tools provided in the institutions might enhance user skills and encourage a more cautious examination of health information accessible on the internet [10].

In our study, 38.61% read about monkeypox disease in their medical school, and only 17.82% attended the educational class teaching about monkeypox disease conducted by their medical college or hospital. In the study by Alshahrani et al. in Saudi Arabia, only 38.2% of participants received training programs about monkeypox [9], which was similar to our result. Both studies conducted in different geographic locations in India and Saudi Arabia showed inadequate training on monkeypox by medical personnel in medical schools and hospitals and showed the necessity of improving this to increase the knowledge of the disease among medical personnel.

Participants' perception of their own knowledge of monkeypox was determined in our study on a scale of 1-10. Two hundred and six (50.99%) participants scored their knowledge on a scale of 1-5, and 198 (49.01%) participants scored their knowledge on a scale of 6-10.

The participants were asked 20 questions to assess their knowledge of monkeypox. The average correct responses of participants who perceived to have poor knowledge were significantly lower than the participants who perceived to have good knowledge, (p-value of 0.0004).

In our study, the participant's perception of their knowledge of monkeypox was proportional to the test scores. In a study conducted by Sallam et al., knowledge about monkeypox was significantly and independently linked with conspiracy theories associated with the emerging virus. It was observed that a lower understanding of monkeypox was correlated with a greater number of conspiracy theories [6]. As a result, adding courses to the curriculum that discuss new infectious diseases of medical and other health-related facilities/schools using modern instructional technology in an innovative way might be advantageous to monitor conspiracy beliefs by enhancing the knowledge of the diseases [11]. At least in modeling experiments done by Brainard et al., it has been demonstrated that reducing misinformation in outbreak scenarios enhances the outcomes for public health [12].

Several studies determine the medical personnel's confidence in managing monkeypox. It is evidenced that training in healthcare facilities boosts healthcare personnel's confidence in managing ailments [13,14]. Receiving knowledge about monkeypox during medical school was the only factor associated with general practitioners' confidence, and it raised the odds of having a favorable opinion by about three times, according to a study by Harapan et al. that examined the confidence when handling monkeypox in humans among medical professionals in Indonesia. Attendance at national meetings and length of practice were other determinants of the doctor's confidence [15].

Our study had several limitations. The questionnaire was circulated online using various social media websites. Medical students, interns, or recent graduates with active social media presence and internet access, and those in contact with authors, were more likely to respond to the questionnaire than others who may not have received it. Even though the authors belong to different medical schools in different parts of India. Another limitation is that those who are more interested in the topic of monkeypox disease or have read about it are more likely to respond. Lastly, the sample size is small, and further studies should be conducted with a bigger sample size and should be carried over several days to reach as many participants as possible. 

## Conclusions

The study shows that the majority of the information about monkeypox was obtained from online sources. Almost an equal proportion of participants perceived to have poor and good knowledge about monkeypox. Assessment of the knowledge of monkeypox revealed that the average correct responses of participants who perceived to have poor knowledge were significantly lower than the participants who perceived to have good knowledge. A higher level of knowledge is vital in eliminating misinformation or conspiracies about the disease will positively impact public health. Knowledge of diseases gained via medical education in medical school, length of clinical practice, and attendance at national conferences greatly boost physicians' confidence in tackling emerging infectious diseases like monkeypox.

## Appendices

Questionnaire

1. Name (optional)

2. Age:

3. Gender:

4. Select the best: Medical student / Intern / Graduate

5. Have you read about monkeypox disease in your medical school? Yes / No

6. Did your medical school/hospital conduct any educational classes to teach about monkeypox disease?

7. If yes, did you attend it? Yes / No

8. Have you recently read about monkeypox disease (past 1 year) from any textbook or online resource? Yes / No

9. If yes, then select the best: Textbook or Online or Newspaper

10. If online, select the best: WHO website / UpToDate / Other website / Instagram / LinkedIn / Twitter / Facebook

11. Do you keep yourself updated on the latest information about monkeypox disease? Yes / No

12. On a scale of 1-10, how much do you think you know about monkeypox disease? 

13. Do you think there is a need to provide more educative materials to medical students, interns, and graduates to make them aware of monkeypox disease? Yes / No

**Table 6:** Questions about monkeypox

Question	Response	
1. The first human case of monkeypox was discovered in 1970.	Yes	No
2. The recent outbreak of monkeypox cases was seen in May 2022.	Yes	No
3. In the recent outbreak, the initial cluster of cases was reported in the United States.	Yes	No
4. Inoculation of the virus to skin and mucosal surfaces occurs by direct contact	Yes	No
5. Monkeypox virus replicates at the site of inoculation	Yes	No
6. The incubation period lasts from four to five days.	Yes	No
7. During the incubation period, the monkeypox virus is contagious and highly infectious	Yes	No
8. Manifestations of the monkeypox virus can be seen in the prodromal stage	Yes	No
9. Monkeypox is a sexually transmitted infection	Yes	No
10. The majority of reported monkeypox cases have a travel-related link to an endemic country	Yes	No
11. Most monkeypox cases have been among people who identify as men who have sex with men	Yes	No
12. Monkeypox patients also have a sore throat along with a fever	Yes	No
13. Monkeypox patients present with skin lesions	Yes	No
14. The vesicles are incredibly painful	Yes	No
15. Many monkeypox patients present with genital pain, rectal pain	Yes	No
16. Treatment of monkeypox is mostly symptomatic	Yes	No
17. Two antiviral drugs that may be used for monkeypox infections are tecovirimat and brincidofovir	Yes	No
18. Post-exposure vaccination can also be given to patients who have had close contact with an individual infected with the monkeypox virus	Yes	No
19. Smallpox vaccination provides cross-protection against monkeypox	Yes	No
20. Vaccination is given within four days of exposure to prevent disease or up to 14 days after exposure to reduce the severity of the disease	Yes	No

## References

[1] Petersen E, Kantele A, Koopmans M, Asogun D, Yinka-Ogunleye A, Ihekweazu C, Zumla A (2019). Human monkeypox: epidemiologic and clinical characteristics, diagnosis, and prevention. Infect Dis Clin North Am.

[2] Hraib M, Jouni S, Albitar MM, Alaidi S, Alshehabi Z (2022). The outbreak of monkeypox 2022: an overview. Ann Med Surg.

[3] Harapan H, Setiawan AM, Yufika A (2020). Knowledge of human monkeypox viral infection among general practitioners: a cross-sectional study in Indonesia. Pathog Glob Health.

[4] Thornhill JP, Barkati S, Walmslet S (2022). Monkeypox virus infection in humans across 16 countries - April-June 2022. N Eng J Med.

[5] McCollum AM, Damon IK (2014). Human monkeypox. Clin Infect Dis.

[6] Sallam M, Al-Mahzoum K, Dardas LA (2022). Knowledge of human monkeypox and its relation to conspiracy beliefs among students in Jordanian health schools: filling the knowledge gap on emerging zoonotic viruses. Medicina.

[7] van Mulukom V, Pummerer LJ, Alper S (2022). Antecedents and consequences of COVID-19 conspiracy beliefs: a systematic review. Soc Sci Med.

[8] Muğaloğlu EZ, Kaymaz Z, Mısır ME, Laçin-Şimşek C (2022). Exploring the role of trust in scientists to explain health-related behaviors in response to the COVID-19 pandemic. Sci Educ.

[9] Alshahrani NZ, Mitra S, Alkuwaiti AA (2022). Medical students' perception regarding the re-emerging monkeypox virus: an institution-based cross-sectional study from Saudi Arabia. Cureus.

[10] Battineni G, Baldoni S, Chintalapudi N, Sagaro GG, Pallotta G, Nittari G, Amenta F (2020). Factors affecting the quality and reliability of online health information. Digit Health.

[11] Cervantes J (2020). The future of infectious diseases education. Med Sci Educ.

[12] Brainard J, Hunter PR (2020). Misinformation making a disease outbreak worse: outcomes compared for influenza, monkeypox, and norovirus. Simulation.

[13] White CB, Kumagai AK, Ross PT, Fantone JC (2009). A qualitative exploration of how the conflict between the formal and informal curriculum influences student values and behaviors. Acad Med.

[14] Lönnbro J, Nylén K, Wallerstedt SM (2019). Developing professional confidence in the art of prescribing-a randomized controlled study on structured collegial discussions during internship. Eur J Clin Pharmacol.

[15] Harapan H, Setiawan AM, Yufika A (2020). Confidence in managing human monkeypox cases in Asia: a cross-sectional survey among general practitioners in Indonesia. Acta Trop.

